# Efficacy of early warning signals and spectral periodicity for predicting transitions in bipolar patients: An actigraphy study

**DOI:** 10.1038/s41398-021-01465-w

**Published:** 2021-06-07

**Authors:** Yoram K. Kunkels, Harriëtte Riese, Stefan E. Knapen, Rixt F. Riemersma - van der Lek, Sandip V. George, Arie M. van Roon, Robert A. Schoevers, Marieke Wichers

**Affiliations:** 1 University of Groningen, University Medical Center Groningen, Groningen, Department of Psychiatry, Interdisciplinary Center Psychopathology and Emotion regulation (ICPE), Groningen, The Netherlands; 2 Department of Neurology, Leiden University Medical Centre (LUMC), Leiden, The Netherlands; 3 Department of Vascular Medicine, University of Groningen, University Medical Center Groningen, Groningen, The Netherlands

**Keywords:** Human behaviour, Bipolar disorder

## Abstract

Early-warning signals (EWS) have been successfully employed to predict transitions in research fields such as biology, ecology, and psychiatry. The predictive properties of EWS might aid in foreseeing transitions in mood episodes (i.e. recurrent episodes of mania and depression) in bipolar disorder (BD) patients. We analyzed actigraphy data assessed during normal daily life to investigate the feasibility of using EWS to predict mood transitions in bipolar patients. Actigraphy data of 15 patients diagnosed with BD Type I collected continuously for 180 days were used. Our final sample included eight patients that experienced a mood episode, three manic episodes and five depressed episodes. Actigraphy data derived generic EWS (variance and kurtosis) and context-driven EWS (autocorrelation at lag-720) were used to determine if these were associated to upcoming bipolar episodes. Spectral analysis was used to predict changes in the periodicity of the sleep/wake cycle. The study procedures were pre-registered. Results indicated that in seven out of eight patients at least one of the EWS did show a significant change-up till four weeks before episode onset. For the generic EWS the direction of change was always in the expected direction, whereas for the context-driven EWS the observed effect was often in the direction opposite of what was expected. The actigraphy data derived EWS and spectral analysis showed promise for the prediction of upcoming transitions in mood episodes in bipolar patients. Further studies into false positive rates are suggested to improve effectiveness for EWS to identify upcoming bipolar episode onsets.

## Introduction

Patients diagnosed with bipolar disorder (BD) suffer from recurrent episodes of depression and mania, interchanged with stable or euthymic periods^1^. Rapid transitions in mood, behavior, psychomotor agitation, and sleep may occur^2–4^ with a debilitating impact on patients and their families. One of the key goals of treatment is to maintain euthymic state and prevent relapse. Ideally, treatment is tailored to counter upcoming symptom transitions but both patients and clinician are often late in signaling that a new episode is developing. A promising approach to foresee upcoming transitions comes from complex systems literature, which suggests that a set of generic early-warning signals (EWS) could identify whether resilience to change is declining. Such decreasing resilience can suggest that a transition from one state to another is forthcoming, for example, in global financial markets, biological phenomena, and ecological systems^5^.

Examples of commonly used EWS include rising *variance*, *autocorrelation*, and *kurtosis*^5–7^. Variance indicates how much values of interest deviate from the mean and each other, autocorrelation indicates how similar a variable is to a delayed copy of itself, and kurtosis informs on the shape of the probability distribution. It was shown that these three EWS substantially increased right before a transition^5^. As EWS are generic they do not depend on specific contexts or topics, and are expected to perform similarly within different complex systems. If the typical BD characteristics of transitions towards either manic or depressive episodes also behave as a complex system, detection of EWS may provide a new approach to foresee these transitions. In this conceptualization, we expect the transitions towards a bipolar episode are indicated by the proximity of so-called tipping points. At these tipping points critical slowing down is expected to occur; meaning that the dominant eigenvalue corresponding to recovery rate will go to zero^5^. Such events can—under the right circumstances—be identified by declining resilience indicators, such as the aforementioned EWS.

Practically, we would thus expect to find increased variance, autocorrelation and kurtosis, before the onset of a bipolar episode. Moreover, we speculate that mean physical activity levels could help differentiate between whether the episode change predicted by EWS is either manic or depressive in nature. We expect to find higher levels of physical activity before patients enter a manic episode and lower activity levels before a depressed episode.

Besides EWS, other more content-specific indices might also facilitate prediction of upcoming episodes, such as psychomotor-oriented indices. A growing number of studies are unraveling the deregulation of the circadian sleep/wake cycle by analyzing psychomotor agitation and sleep disturbances -symptoms typically seen in Bipolar patients^2,8^. For example, Bipolar patients showed increased sleep duration, less daytime sleep, and larger contrast between day and night activity during their euthymic period compared to their manic/mixed episodes^8,9^. Moreover, compared to controls, Bipolar patients showed a more fragmented sleep/wake cycle as indicated by more variability within days and less stability over multiple days in their actigraphy data^10^. A related finding is that variability in the sleep/wake cycle can be indicative for the upcoming onset of a depressive episode among bipolar patients during their euthymic periods^11^. A analysis method well suited for studying such changes in the sleep/wake cycle is *spectral analysis;* a technique wherein the variation in the time domain of time-series data is decomposed in their respective frequencies^12^. Consider, for example, actigraphy data of a healthy individual. Healthy humans have a near 24-h sleep/wake cycle, so their spectrum will show the largest peak close to the 24-h frequency even under atypical circumstances such prolonged isolation from natural external Zeitgebers^13^. Hence, deviations from the 24-h frequency may indicate disturbances in the sleep/wake cycle, as for example a 48-h sleep/wake cycle was detected prior to a transition from a depressed state into a manic state in bipolar patients^14^. Disturbances in spectral periodicity are expected to be indicative of increased risk for transition to a manic or depressive episode as they signal dysregulation of the sleep/wake cycle; a feature observed in bipolar patients.

Given the impact of disturbances in activity and the sleep/wake cycle on mood episodes in bipolar patients, actigraphy is especially suited to investigate such upcoming transitions. However, while the aforementioned studies on this topic offer useful hypothesis generating information, only short-term actigraphy time-series data or questionnaires were used. Moreover, none of the aforementioned studies included live transitions in mood episodes while being monitored with actigraphy. As such, prior conclusions can be considered to be limited regarding the effects of sleep/wake disturbances on mood transitions. To study whether changes in EWS predict upcoming manic or depressive episodes in bipolar patients, a study design in which patients are monitored for multiple weeks is required. Actigraphy can be employed to continuously assesses time-series data of physical activity from which onsets of episodes could be predicted. Such continuous activity measurements are better suited than short-term activity measurements to fully capture some of the hallmark symptoms of BD, namely the disturbances in activity and sleep/wake cycles over time. In actigraphy studies, patients wear lightweight, wireless, wrist-worn accelerometers which measure (tri-axial) movement. Actigraphy is relatively simple and has been successfully validated against polysomnography for predicting sleep/wake cycles^15^. As such, its ease of use and its objective nature make actigraphy well suited for monitoring patients with bipolar disorder.

To investigate how transitions towards the onset of either a depressive or manic episode in Bipolar patients can be predicted, we will examine whether declining resilience—as indicated by the EWS—can help anticipate such transitions. Moreover, we will investigate whether disturbances in the periodicity of the sleep/wake cycle can also aid in predicting such transitions. Actigraphy data collected for six months by Bipolar patients will be used. We hypothesize that in the period before the onset of *either* a manic or depressive episode, actigraphy patterns of Bipolar patients will show: (1) rising variance, (2) rising kurtosis, (3) rising autocorrelation in actigraphy activity patterns, and (4) spectral indices indicating disturbances in the typical 24-h wake/sleep cycle. Moreover, we hypothesize that the period before an episode patients will show: (5) mean activity levels congruent with the type of episode (i.e., finding higher mean levels of activity before a manic episode, and lower mean levels of activity before a depressive episode). Our study was pre-registered, meaning that we disclosed our hypotheses and analysis plans before conducting the study and before looking at the data for meaningful patterns, thereby optimizing transparency and replicability. Moreover, we endeavored to make our materials and procedures as open as possible by storing these on a publicly accessible repository (see: https://osf.io/63d8w/).

## Methods and materials

### Sample

An existing dataset of patients diagnosed with BD type I was used for the current study. Details of the current data collection are described elsewhere^16^. Patients were mainly enrolled from the outpatient clinic of the University Center Psychiatry (UCP) within the University Medical Center Groningen (UMCG), and secondarily from the Dutch patient society (“Plusminus”). The inclusion criteria were: (1) diagnosed with bipolar disorder type I, (2) having suffered from at least 1 episode in the past 2 years, and (3) being motivated to participate in a long-term study. The exclusion criteria were: (1) suffering from somatic diseases which could interfere with the actigraphy measurements, and (2) suffering from somatic sleep disorders, such as for example sleep apnea. Patients participated in 180 days of mood monitoring once a day, weekly symptom monitoring, and continuous activity monitoring. In total, 15 patients provided informed consent for their participation in the study. Of these 15 patients, one dropped out during the study due to personal reasons, whilst the data of one patient was found to be largely incomplete and had to be excluded as well. Of the 13 remaining patients, 11 did experience a transition towards a mood episode (4 experienced a manic episode and 7 experienced a depressive episode). The two patients who did not show any transition towards a mood episode during the study period were excluded. Lastly, of the remaining 11 patients three experienced so many mood symptoms that their designated “euthymic” episode was not evidently euthymic anymore. These patients were thus excluded from the analyses. Eight patients were included in the final sample.

### Symptom indices

Depressive and manic episodes were defined with validated questionnaires. Every week patients filled out the Inventory of Depressive Symptomatology - Self Rating (IDS-SR^17^), and the Altman Self Rating Scale for Manic symptoms (ASRM^18^). In order to be designated as being in a manic episode, patients had to score higher than 5 points on the ASRM for two consecutive weeks, assuring that there is at least one full week of manic symptoms present. The criteria of scoring at least 5 points on the ASRM for two weeks was included because ASRM ratings can reflect the mental state on the day of completing the ASRM more than the mental states of several days before^19^. Depressive episodes, on the other hand, required patients to maintain a score higher than 25 points on the IDS-SR for at least three consecutive weeks, assuring that there are at least two full consecutive weeks of symptoms. A transition was defined as starting to fulfill the aforementioned criteria for depressive and manic episodes. For our analyses, we studied the first transition of each patient.

### Activity

Actigraphy time-series data were collected with a wrist-worn MotionWatch 8 (MW8; CamNTech) actigraph. Patients were instructed to wear the MW8 continuously, only removing the device under rare conditions, such as sauna visits where a combination of high humidity and temperature could induce technical difficulties. The MW8 was initialized to use one-minute epoch lengths, no data compression, and no light detection. An electronic sleep diary was filled-out every morning.

### Analyses

Pre-processing and analysis of the data was performed in the statistical programming language R^20^. For pre-processing and analyzing the actigraphy data, the R package *ACTman*^21^ was used. Generic EWS such as autocorrelation and kurtosis, are sensitive to detrending^7^. Therefore, we removed linear trends from the data by calculating the least squares regression line to estimate the growth rate, and then subtract differences from the least squares fit line from the data. For the spectral analysis, we calculated the spectral periodogram with a fast Fourier transformation without smoothing in R statistical software^20^.

Three EWS indices were calculated from 1 min actigraphy time-series averages for each participant independently. Variance and kurtosis were calculated over the minute-level actigraphy data in a moving window. We used a window size of 7 days which means that every window includes at least one Saturday and one Sunday, thereby equalizing any possible effects from weekend days. The size of the steps at which the moving window was moved over the data was set to one day; the algorithm first calculated the EWS for the first 7-day window, then moved the window 1 day ahead, and repeated these steps until the last full window was calculated. Autocorrelation indicates how much a variable correlates with itself at a later lagged instance of itself. Based on conceptual reasoning, we chose to investigate autocorrelation at lag 720 min (acf-720). Autocorrelations at lag-720 are informative about how activity is correlated to the amount of activity 12 h (=720 min) earlier: for example, the correlation between the amount of activity at 12:00 to the amount of activity at 00:00 midnight. The autocorrelation of activity separated by 12 h is expected to be negative, whereas autocorrelation of activity separated by 24 h is expected to be positive. However, if the normal sleep/wake cycle gets deregulated and the contrast between sleep and waking hours diminishes, we expect acf-720 to approximate zero or even positive values; either due to an individual becoming more restless during sleeping hours (an expected symptom of a manic episode), or by getting less active during waking hours (an expected symptom of a depressive episode).

We investigated whether strong increases in EWS occur up till four weeks before the onset of an episode. This four-week period was chosen to allow for a plausible extent of time for increases in EWS to develop form. To test whether the increase in EWS is significant the *Mann-Kendall* test^22,23^, a commonly used non-parametric test for detecting significant monotonic trends in time-series data, was used. In order to estimate disturbances in spectral periodicity we examined the ratio between the fundamental and second harmonic frequencies. Here, the fundamental frequency represents the lowest frequency of a periodic waveform, whereas the harmonic frequencies are frequencies that operate at (whole-number) multiples of the fundamental frequency. The ratio between these two estimates the likeliness that a disturbance in periodicity is afoot as it indicates the strength of the fundamental 24-h frequency versus the strength of an alternative frequency (the 12-h frequency in this case). The ratio between the fundamental and second harmonic frequencies was calculated by dividing the power spectral density value of the second harmonic by the same the fundamental frequency. Harmonic frequencies were calculated by one divided by *n* times the fundamental period. Lastly, for the mean activity level analyses we considered 7-day periods around the onset of the episode, operationalized as starting one week before episode onset. These mean levels were then compared with mean activity levels 7-day periods from the euthymic period.

## Results

### Early-warning signals

**Fig. 1:**
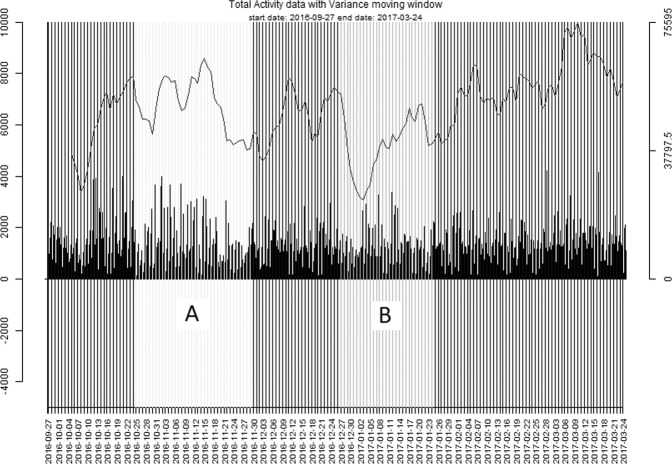
Variance marker of patient 1, calculated from a moving window of the activity data (see method section for details). The left set of light-gray vertical lines indicated with an “A” represent the euthymic period. The right set of light-gray vertical lines indicates with a “B” represent the episode period. The y-axis shows the variance of the activity while the x-axis represents time in days.

Results are presented in Table 1, and plots of the EWS for all patients can be found on an online repository (see: https://osf.io/63d8w/). An example of an EWS which significantly increased before an episode is given in Fig. 1. In one patient (ID 11) the transition occurred so early during data collection that only three weeks of data are available before the transition. Mann-Kendall trend tests showed significant trend increases in EWS up till four weeks before the onset of either a manic or depressive episode in seven patients. In two patients, their episode onset was preceded by significant changes in all three EWS. In five patients, their episode onset was preceded by significant changes in acf-720 only. In one patient, the episode onset was preceded by significant increases in variance only. Lastly, in one patient, the episode onset was preceded by significant increases in both variance and kurtosis. However, although the direction of the change was always in the expected direction for variance and kurtosis (i.e. increasing), for acf-720 it significantly decreased in four patients preceding episode onset, while in one patient it significantly increased. Finding effects in the opposite direction for acf-720 might suggest that it operates more like a general measure of instability, wherein any change from the normal rhythm is indicative of an upcoming episode, not necessarily only increases. Post-hoc analyses using Fisher’s test for combined p-values were performed for each EWS to investigate whether overall significant trends were present. By combining the *p*-values of each EWS, outcomes are less dependent on the individual patient and should thus be more generalizable. Fisher’s test for combined p-values is performed by taking the *p*-values for one indicator and from this calculate the chi-values. Lastly, taking one minus the calculated chi-squared cumulative density distribution using a transformation to *N*(0,1) will yield the required combined group p-values. Group p-values obtained via this method were (1) Variance, group *p-value* < 0.001, (2) Kurtosis, group *p-value* < 0.001, (3) Acf-720, group *p-value* < 0.001. This suggests that the individual quantifiers were significant overall, and that the three quantifiers are reliable indicators of upcoming transitions.

**Table 1:** Outcomes of the mann-kendall trend tests for ews assessment.

ID	Episode type	Early warning signal	z-scores	tau	n	p-values	Direction
1	D	Variance	3.319	0.444	28	0.001**	increase
1	D	Kurtosis	1.857	0.249	28	0.066	increase
1	D	Acf-720	1.663	0.233	28	0.096	increase
2	M	Variance	0.988	0.132	28	0.336	increase
2	M	Kurtosis	0.553	0.074	28	0.597	increase
2	M	Acf-720	−2.736	−0.380	28	0.006**	decrease
3	D	Variance	0.909	0.122	28	0.377	increase
3	D	Kurtosis	−1.976	−0.265	28	0.05	increase
3	D	Acf-720	−5.719	−0.783	28	< 0.001**	decrease
4	M	Variance	2.015	0.270	28	0.045*	increase
4	M	Kurtosis	−3.714	−0.497	28	0.001**	increase
4	M	Acf-720	2.894	0.419	28	0.004**	increase
8	M	Variance	−3.161	−0.423	28	0.001**	increase
8	M	Kurtosis	2.134	0.286	28	0.034*	increase
8	M	Acf-720	−2.341	−0.328	28	0.019*	decrease
9	D	Variance	0.198	0.026	28	0.860	increase
9	D	Kurtosis	1.936	0.259	28	0.055	increase
9	D	Acf-720	−3.935	−0.553	28	< 0.001**	decrease
11	D	Variance	0.121	0.019	21	0.929	increase
11	D	Kurtosis	0.846	0.133	21	0.420	increase
11	D	Acf-720	−0.799	−0.132	21	0.424	decrease
15	D	Variance	3.161	0.423	28	0.001**	increase
15	D	Kurtosis	−3.082	−0.413	28	0.002**	increase
15	D	Acf-720	−1.013	−0.139	28	0.311	decrease

Note: **: *p* < = 0.01; * *p* < = 0.05; episode type indicates whether a depressive episode (D) or a manic episode (M) emerged; Details on calculation of the 3 early warning signal indices (Variance, kurtosis, Acf-720) are given in the method section; Participant ID 11 has less than 4 weeks of data due to their transition occurring early during data collection.

### Disturbances in periodicity

**Fig. 2:**
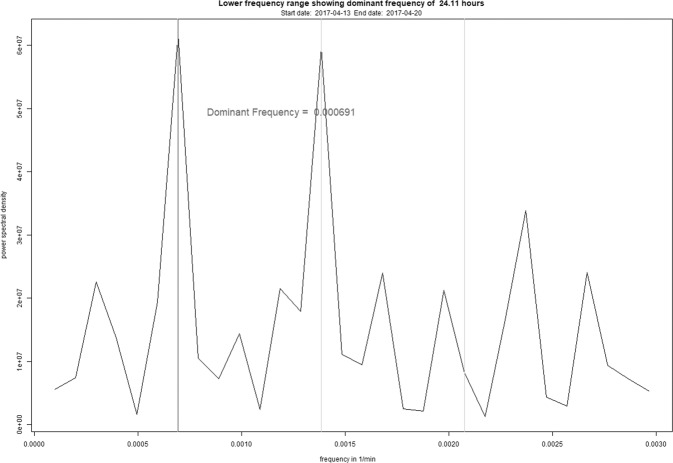
Frequency spectrum of patient 11 showing the dominant frequency (dark gray) and its two subsequent harmonic frequencies (light gray), one day before transitioning towards an atypical 12-h rhythm. The y-axis shows the power spectrum density in U^2^ while the x-axis shows the frequency in 1/min.

**Fig. 3:**
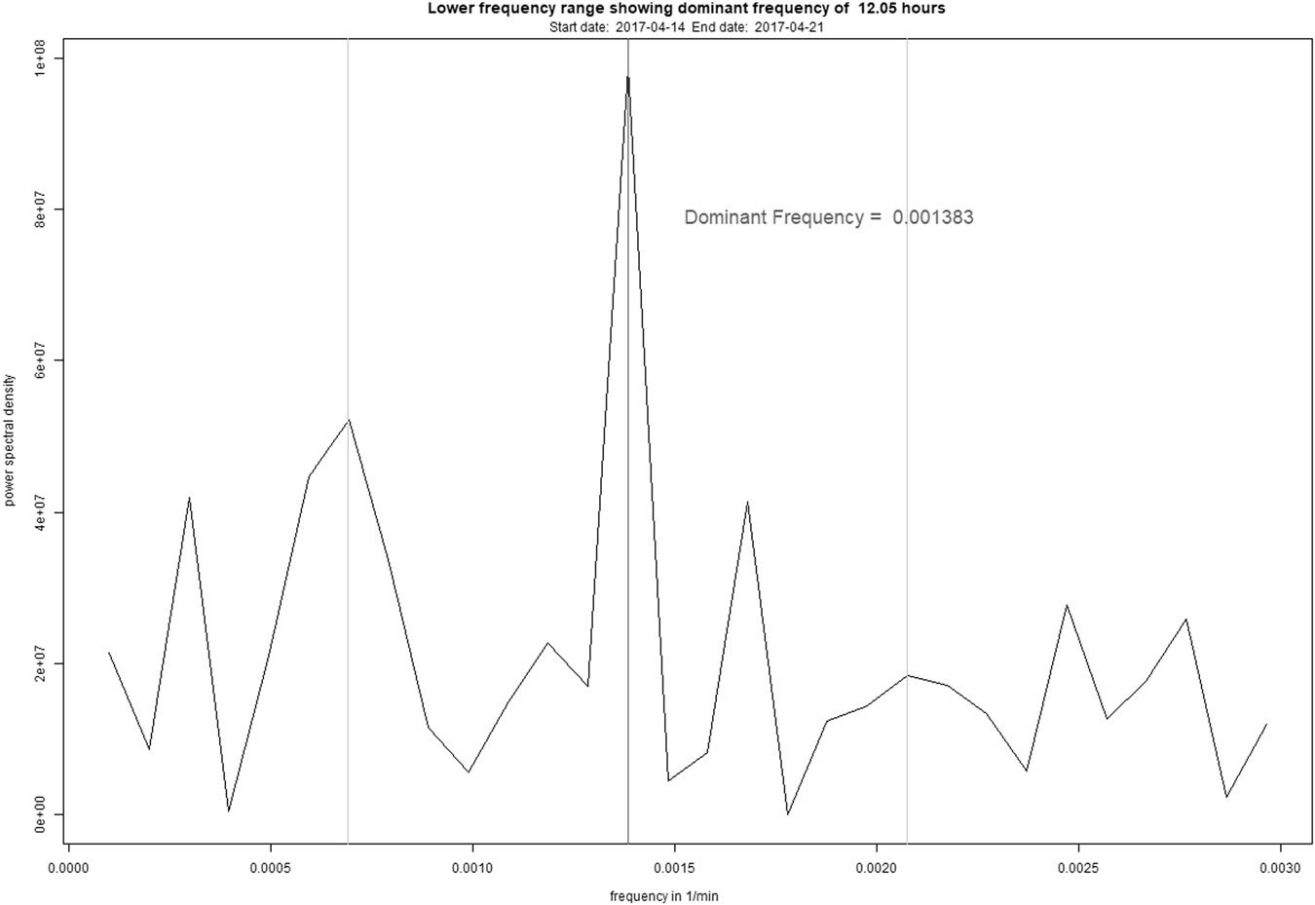
Frequency spectrum of patient 11 showing the new dominant frequency (dark gray) after transitioning from a typical 24-h rhythm to an atypical 12-h rhythm. The y-axis shows the power spectrum density in U^2^ while the x-axis shows the frequency in 1/min.

In one patient (no. 11) the *onset* of an episode was preceded by the hypothesized rhythm transition and the increased frequency ratio associated with it. This change in periodicity was observed nine days before the start of the episode. During that day the ratio between the fundamental frequency and its second harmonic reached a value of 0.98, which indicates an upcoming change from a 24-h rhythm to an atypical 12-h rhythm, as presented in Figs. 2 and 3. None of the other patients showed the hypothesized change.

Post-hoc exploratory analysis of the spectral results showed that one other patient (no. 3) also showed similar disturbances in periodicity, albeit from a 24 h rhythm towards an atypical 4-h rhythm. This change in periodicity preceded episode onset by 28 days. Moreover, next to the aforementioned rhythm transition of patient 11, this patient showed another transition wherein the *end* of the *euthymic period* was preceded by a change in periodicity. Here, the periodicity change preceded the end of the euthymic period by 17 days, while the ratio between the fundamental frequency and the second harmonic reached a value of 0.96. Here the ratio between the fundamental frequency and the second harmonic already approximated one, five days before the end of the episode. From these results it may be concluded that the used spectral periodicity indices act more as a general indicator of instability in mood than a specific indicator for one particular type of transition.

### Mean activity levels

The results of mean level changes in anticipation of the defined transition episodes are presented in Table 2. All patients who developed a depressive episode showed higher activity levels during the first seven days before their episode when compared to the first seven days of their euthymic period. One of the three patients who developed a manic episode showed less activity during the first seven days before entering a manic episode when compared to the first seven days of their euthymic period. In six of the eight patients mean activity levels did not develop in the expected direction and thus, did not contribute to the differentiation whether an upcoming episode is manic or depressive in nature.

**Table 2:** Mean activity during seven-day periods before bipolar episodes and during euthymic periods.

ID	Episode type	Mean euthymic	Mean episode	Interpretation
1	D	96.1	139.1	More activity before episode
2	M	153.8	124.6	Less activity before episode
3	D	100.4	109.1	More activity before episode
4	M	51.2	61.7	More activity before episode
8	M	91.9	92.9	More activity before episode
9	D	171.3	211.0	More activity before episode
11	D	186.2	192.0	More activity before episode
15	D	110.7	166.2	More activity before episode

Note: episode type indicates whether a depressive episode (D) or a manic episode (M) emerged; mean actigraphy values are given in *MotionWatch count* units.

Post-hoc exploratory analyses were performed to ascertain that the aforementioned results were not caused by artefacts due to mean levels of activity *during* episodes that were in the expected direction. After correcting for this, five of the eight patients showed an effect in the expected direction; three showed less activity during their depressed episode than in their euthymic period, and two showed more activity during their manic episode than in their euthymic period (see Table 3).

**Table 3:** Mean activity during periods before bipolar episodes.

ID	Episode type	Mean euthyic	Mean episode	Interpretation
1	D	108.403	87.236	Less activity during episode
2	M	145.258	144.386	Less activity during episode
3	D	94.089	96.359	More activity during episode
4	M	49.156	54.576	More activity during episode
8	M	94.559	112.914	More activity during episode
9	D	196.669	196.399	Less activity during episode
11	D	186.464	168.594	Less activity during episode
15	D	121.871	137.651	More activity during episode

Note: episode type indicates whether a depressive episode (D) or a manic episode (M) emerged; mean actigraphy values are given in *MotionWatch count* units.

## Discussion

In the current study, we applied generic *Early-Warning Signals* (EWS) and spectral periodicity analysis calculated from actigraphy time-series data to investigate whether we could predict upcoming mood transitions in patients suffering from bipolar disorder (BD). We tested whether three EWS (i.e. variance, kurtosis, and autocorrelation at lag-720), showed significant changes up till four weeks before the onset of a manic or depressive episode. We found that in seven out of eight patients a significant change in at least one of these three EWS could be identified up till four weeks before the onset of an episode. For the variance and kurtosis EWS, the effect was in the expected direction, thereby confirming our first two hypotheses. For the acf-720 EWS, the effect was in the expected direction in one patient, but in the opposite direction in four patients, thereby rejecting our third hypothesis. Such a result suggests that acf-720 seems to act as a more general EWS which can signal both increases and decreases, which implies that shifts in either direction can precede episode. Acf-72 was able to detect episode onsets in three patients that the variance and kurtosis EWS did not pick up. Moreover, when considering more large scale trends, both increases and decreases could be observed. Yet, our finding that autocorrelation effects were often in the opposite direction of what was expected do cast doubt on whether the observed transitions in bipolar patients are best described and predicted with zero-eigenvalue tipping points. For the fourth hypothesis we expected to find disturbances in the typical 24-h sleep/wake cycle before the onset of an episode. However, the hypothesized pattern was only observed in one out of eight patients. We thus rejected our fourth hypothesis. Our fifth hypothesis concerned testing whether mean activity levels are congruent with the episode type before the start of the episode. We have rejected this hypothesis as only two out of eight patients showed the expected effect. Nonetheless, post-hoc analyses showed mean activity levels congruent with the episode type when data during the episode was analyzed instead of data from before the episode.

The EWS results did support the hypothesis from complex system theory that actigraphy derived EWS, did precede transitions such as onsets of bipolar disorder episode onset. Commonly used EWS such as variance and kurtosis seem to operate complementary to more context-driven EWS, such as autocorrelation at lag-720. These findings suggest that a combination of effective, personalized EWS could be potential useful in clinical practice. Such a clinical tool could be used to monitor a patient’s risk for developing a clinical relevant manic or depressive episode, and warn clinicians in time to temper or even prevent upcoming BD episodes. As this clinical tool used actigraphy data, it offers patients a low burden method to monitor BD episode risk. Moreover, more research is required to assess the effectiveness of these, and other EWS in larger samples to investigate whether more effective combinations of EWS can be found. Here combining an actigraphy-based method with subjective time-series data, derived with the experience sampling method, may be the next step forward to enhance BD episode risk assessment.

Regarding the spectral indices, we found that the investigated spectral periodicity indices appeared to be somewhat sensitive to multiple types of transitions (amongst others, towards the *start* of the episode or towards the *end* of the *euthymic period*). However, results were not as clear-cut as found in earlier studies, wherein for example, distinct 48-h sleep/wake cycles were found in bipolar patients who transitioned from depressed to manic episodes^14^. Here, the spectral periodicity indices thus behaved more like general mood instability indicators than as an exclusive indicator of *episode onsets*†.

Despite the innovative character of our study, there are a number of issues that need to be addressed. First, pre-collected data were used, whereas only performing the analysis while the patient is still monitoring herself would allow for real-time detection of changes in BD episodes. However, there is a current lack of actigraphy hardware that can send information in real-time for long-term continuous monitoring and calculation of indices. Therefore, extensive cooperation between researchers, clinicians, patients, and actigraphy hardware manufacturers is needed to develop the infrastructure required for such real-time monitoring of bipolar patients. Second, another issue is the relatively low number of patients in our sample. Yet, this is somewhat offset by the relatively large number of data points collected by each patient (1440 observations each day, for approx. 180 consecutive days). While the large number of observations does offer confidence in the robustness of the within patient findings, a replication study with a larger number of patients for the same time period could provide improved generalizability of the obtained results. Third, the current study did not investigate the *false positive rate*, the number of times the EWS would have falsely suggested that a transition is afoot, while in fact none is. As such false negatives can reduce the effectiveness of EWS as a clinical tool, this point should be investigated further in future studies. Fourth, although both variance and autocorrelation are promising resilience indicators for upcoming critical transitions, variance was found to be not as robust as autocorrelation^23^. That is, when environmentally triggered changes that affect the equilibrium of a system itself was found to be able to decrease rather than to increase before an upcoming transition. This lower robustness for variance will be more profound if the system’s own rate of change is relatively slow when compared to the frequency of the environmentally triggered changes. Fifth, as our data is time-correlated the obtained trends in the indicators could be due to chance. Bootstrapping is typically a viable strategy to prevent such chance findings^7,24^. However, as the sleep/wake cycle of physical activity introduces a strong daily periodicity in our data, commonly used bootstrapping methods would not be suitable^25^. Given the intricacies of selecting and performing a bootstrap strategy suitable for the current data, such additional analyses would be beyond the scope of this work. Yet we do recognize that a suitable bootstrap analysis in for example a future study, could provide additional evidence for the hypotheses investigated in the current study.

Development and application of EWS in the field of psychiatry is still quite novel. Future studies could aim to elucidate basic EWS properties in psychiatric samples by aiming to answer fundamental questions relevant for the search of EWS such as: *(1) “At what time scale do changes occur in this psychiatric sample?”, (2) “What exact marker (actigraphy, heart rate, mood, etc.) would be best to search for EWS in?”, (3) “How can we help increase the number of n* = *1 studies with large enough samples to establish EWS sensitivity and specificity?”, (4) “Which combination of EWS would outperform most single EWS indices?”, or (5) “How is EWS performance affected by external factors, such as life events or medication use?”*. The answers to these kinds of questions may be helpful to unravel if and how dynamical systems theory fulfills its promise for psychiatric research and implementation in clinical practice.

In summary, we found that both EWS and spectral periodicity indices could facilitate the prediction of upcoming mood episodes in bipolar patients. With the tested EWS we were able to identify upcoming BD episode onsets. Yet, before this method can be used in clinical practice further studies are required to investigate how the tested EWS perform in the absence of transitions; thereby investigating their false positive rates. Context-driven actigraphy based EWS such as autocorrelation at lag-720, require further conceptual study in order to leverage their predictive capabilities, especially regarding their timing, as currently only a period of four weeks was considered. Furthermore, we investigated whether disturbances in periodicity preceded episode onset. Such periodicity disturbances were found to show performance that is more akin to general instability indices than as singular indices for episode onset. While such findings were unexpected, and might be less helpful from a clinical perspective (e.g., when the *end* instead of the *start* of the episode is predicted), they do offer useful theoretical knowledge to assess their effectiveness and limitations in EWS interpretation. The current study thus provides exploratory information on the opportunities and pitfalls of analyzing EWS from actigraphy data. As such, the pioneering work presented in this study can be used as a stepping-stone for future studies examining the possibility to predict mood transitions by using actigraphy data, both in patients suffering from bipolar disorders as well as in other mental disorders.

## References

[1] López-Muñoz F, Vieta E, Rubio G, García-García P, Alamo C (2006). Bipolar disorder as an emerging pathology in the scientific literature: a bibliometric approach. J. Affect Disord..

[2] Harvey A (2011). Sleep and circadian functioning: critical mechanisms in the mood disorders?. Annu Rev. Clin. Psychol..

[3] Jackson A, Cavanagh J, Scott J (2003). A systematic review of manic and depressive prodromes. J. Affect Disord..

[4] Sierra P, Livianos L, Arques S, Castelló J, Rojo L (2007). Prodromal symptoms to relapse in bipolar disorder. Aust. NZ J. Psychiat..

[5] Scheffer M (2009). Early-warning signals for critical transitions. Nature.

[6] Biggs R, Carpenter S, Brock W (2009). Turning back from the brink: Detecting an impending regime shift in time to avert it. Proc. Natl Acad.Sci. USA.

[7] Dakos V (2012). Methods for detecting early warnings of critical transitions in time series illustrated using simulated ecological data. PLoS ONE.

[8] Alloy, L., Ng, T., Titone, M. & Boland, E. Circadian rhythm dysregulation in bipolar spectrum disorders. *Curr Psychiatry Rep*. 2017;19. 10.1007/s11920-017-0772-z 10.1007/s11920-017-0772-z PMC6661150 28321642

[9] Salvatore P (2008). Circadian activity rhythm abnormalities in ill and recovered bipolar I disorder patients. Bipolar Disord..

[10] Jones S, Hare D, Evershed K (2005). Actigraphic assessment of circadian activity and sleep patterns in bipolar disorder. Bipolar Disord..

[11] Ng T, Chung K, Ng T, Lee C, Chan M (2016). Correlates and prognostic relevance of sleep irregularity in inter-episode bipolar disorder. Compr. Psychiatry.

[12] Box, G., Jenkins, G. & Geinsel, G. Time series analysis forecasting and control. New Jersey: Wiley; 2008.

[13] Basner M (2013). Mars 520-d mission simulation reveals protracted crew hypokinesis and alterations of sleep duration and timing. PNAS.

[14] Wehr T (1982). 48-hour sleep-wake cycles in manic-depressive illness. Arch. Gen. Psychiatry.

[15] Pollak C, Tryon W, Nagaraja H, Dzwonczyk R (2001). How accurately does wrist actigraphy identify the states of sleep and wakefulness?. Sleep.

[16] Knapen, S. E. Rhythm & Blues: Chronobiology in the pathophysiology and treatment of mood disorders (Doctoral dissertation, Rijksuniversiteit Groningen, Groningen, the Netherlands). 2019. Retrieved from: https://www.rug.nl/research/portal/en/publications/rhythm--blues(94b2329b-7ff7-44ee-a095-b330d92735a6).html.

[17] Rush A, Gullion C, Basco M, Jarrett R, Trivedi M (1996). The inventory of depressive symptomatology (IDS): psychometric properties. Psychol. Med..

[18] Altman E, Hedeker D, Peterson J, Davis J (1997). The Altman self-rating mania scale. Biol. Psychiatry.

[19] aan het Rot M, Hogenelst K, Schoevers R (2012). Mood disorders in everyday life: a systematic review of experience sampling and ecological momentary assessment studies. Clin. Psychol. Rev..

[20] R Core Team. R: A language and environment for statistical computing. R Foundation for Statistical Computing, Vienna, Austria. 2017. https://www.R-project.org/.

[21] Kunkels, Y. K. et al. ACTman: Automated preprocessing and analysis of actigraphy data. *J. Sci. Med. Sport*. 10.1016/j.jsams.2019.11.009 (2019). 10.1016/j.jsams.2019.11.009 31813761

[22] Mann H (1945). Nonparametric Tests Against Trend. Econometrica.

[23] Dakos V, van Nes E, D’Odorico P, Scheffer M (2012). Robustness of variance and autocorrelation as indicators of critical slowing down. Ecology.

[24] Dakos, V. et al. (2008). Slowing down as an early warning signal for abrupt climate change. IOP Conference Series: Earth and Environmental Science, 6, p.062012. 10.1073/pnas.0802430105 PMC2567225 18787119

[25] Politis, D. N. (2001). Resampling time series with seasonal components. *Frontiers in Data Mining and Bioinformatics*: *Proceedings of the 33rd Symposium on the Interface of Computing Science and Statistics*, *Orange County, California, June 13*–*17*, pp. 619–621. (Proceedings).

